# Prognosis in Heart Cases

**Published:** 1913-07-26

**Authors:** 


### THE SCHOOL DOCTOR.

### VII.—Prognosis in Heart Cases.

In former articles we have laid stress on the desirability of revising the orthodox teaching with regard to heart-strain in school children. It is a fact well known to most school doctors that the dangers of so-called overstrain have been much exaggerated, and that in the vast majority of cases more harm than good is done by excluding from all participation in ordinary school life children who, on examination, are found to have a cardiac murmur. In dealing with the examination of the child's heart (Article I.) we briefly referred to the varieties of murmur that may be met with, and considered their significance; and it now remains to deal, equally briefly, with the question of prognosis.

This question, indeed, has been admirably discussed by Dr. Theodore Fisher in an article contributed to the current number of *School Hygiene* ("Common Cardiac Murmurs in Childhood"). Dr. Fisher's views may not be wholly acceptable to all school doctors, but they certainly deserve every consideration, coming as they do from an established authority who has, moreover, had practical experience of school work for many years. His paper is so ably written, so lucid and temperate in expression, that it will command attention even from those who most fundamentally differ from him in opinion. Prefacing his remarks by pointing out the well-known difficulties in accurately mapping out the cardiac dulness in a child, and in depending upon the cardiac impulse as a sign of dilatation, Dr. Fisher proceeds to deal with the various murmurs. "Murmurs are far too common over the hearts of children to indicate the presence, except in rare instances, of pathological dilatation. . . . The great majority of these murmurs (*i.e.*, murmurs developed during febrile attacks, especially acute rheumatism) disappear." "Whatever view we may take of the causation of pulmonary and systolic murmurs audible in children, we will, I think, all agree that, either occurring alone, or more frequently in combination, they are commonly heard in children and in most instances are of no significance whatever. Curiously enough they do not even appear to bear any very close relation to anæmia. They may be heard in robust and healthy children, while in anæmic and delicate children they may be absent." "Experienced school doctors soon consciously, or perhaps subconsciously, learn to ignore murmurs of a certain type as evidence of abnormality worthy of attention." Dr. Fisher gives a remarkable case of a girl, aged eleven, who was said to have been listless and short of breath since an attack of scarlet fever three months before examination; auscultation evidenced "a systolic murmur of high-pitched character audible at the apex, far into the axilla, and also behind." Five years later this girl was a kitchen-maid, in good health, and on auscultation no trace of the murmur could be detected. An equally striking case cited is that of a boy, aged ten, seen seven weeks after an attack of acute rheumatism, in whom the family practitioner had diagnosed a diseased heart. "A high-pitched systolic murmur was audible at the apex and outwards as far as the mid-axilla." Six years later the murmur had completely disappeared, and the boy was playing football and walking three miles to a technical school every day. In fifty-one cases of chorea which had been under Dr. Fisher's care, he tabulated the results of examination after an interval of three years from the time of the first examination; in twenty an apical systolic murmur had been noted at first and in twelve of these this murmur was not heard at the second examination, though in several instances it had been well marked.

With these findings in view most school doctors, who can also add their own experience to the above, will agree that an apical systolic murmur, even when high-pitched in character and well transmitted outwards, frequently disappears. A basal systolic murmur, in Dr. Fisher's opinion, "we expect to disappear." He sounds a note of warning with regard to the need for carefully differentiating between a pulmonary systolic murmur conducted to the right of the sternum and an aortic systolic murmur for which the former may sometimes be mistaken. Our own view is that the majority of cases of aortic disease diagnosed in school children are cases in which such a mistake has been made, for our experience leads us to the conclusion that aortic disease is extremely rare in children—much rarer, in fact, than hospital experience would lead one to suppose. The frequent error of taking an apical diastolic sound to be evidence of mitral stenosis is another point to which Dr. Fisher draws attention. The so-called third sound is by no means uncommon in children, but it is rarely indeed that it can be taken to be evidence of pathological defects of the heart. A good example is cited in the case of a lad of eleven, in whom such a sound was accompanied by a thrill. Five years later this boy was working on a farm; no diastolic sound was audible, and he was apparently perfectly sound. It is well to bear in mind, too, the fact that emotional causes may produce transient murmurs, of which this diastolic irregularity is perhaps as common as any.

The result of recent examinations of children's hearts, by refined methods of orthodiascopy, light percussion, and tracings, goes far to support Dr. Fisher's conclusions. In view of this fact, school doctors would do well to examine suspected cases two or three times before they give a definite opinion. Where the cardiac lesion is so obvious and so serious that it restricts the child from taking part in the ordinary play and work of his or her schoolmates, the case is obviously one that demands a special school and perhaps special home treatment as well. Where the lesion is well compensated and the child's general health is good, there is no reason for excluding the child or for restricting its activity unduly, although in such cases the parents, the teachers, and the child should be made aware of the condition and warned against the possible dangers of overstrain, which, it may be added, are far greater after school life than during the school-going period. On the other hand, ill-marked murmurs, or those of a doubtful character, need scarcely be commented upon to teacher or parent, although in the interests of all concerned it is well for the school doctor to make out a private "following-up card," and to see the child from time to time in order to satisfy himself that there is no further development. This takes time, but it is well worth the trouble involved.

